# Cervical Spine Osteophyte: A Case Report of an Unusual Presentation

**DOI:** 10.7759/cureus.44762

**Published:** 2023-09-06

**Authors:** Mohammad Al-Jafari, Sarah Abu Tapanjeh, Harith Al-Azzawi, Sura Abu Eid, Huda j Baidoun, Mohammad Abu-Jeyyab, Mohammed Y Sarhan, Hiba Jbara, Alaa Akel

**Affiliations:** 1 College of Medicine, Mutah University, Al-Karak, JOR; 2 College of Medicine, Jordan University, Amman, JOR; 3 Department of Orthopedic Surgery, Hashemite University, Al-Zarqaa, JOR; 4 Department of Neurosurgery, Al-Basheer Hospital, Amman, JOR; 5 Department of Orthopedic Surgery, Mutah University, Al-Karak, JOR

**Keywords:** osteophytosis, cervical ostephytes, diffuse idiopathic skeletal hyperostosis (dish), forestier disease, dysphagia, spinal, osteophyte

## Abstract

Diffuse idiopathic skeletal hyperostosis (DISH) is a condition that causes abnormal bone growth at the sites of ligament insertion, mainly in the spine. It is of unknown etiology and usually affects older males. It is often asymptomatic, but it can sometimes cause dysphagia if it affects the anterior cervical spine.

We report the case of a 50-year-old male patient with DISH who presented with chronic dysphagia and was diagnosed with a large cervical osteophyte compressing the esophagus. The patient had a history of several comorbidities, including diabetes, hypertension, stroke, and gout. He underwent surgical removal of the osteophyte and recovered well. We discuss the clinical features, diagnosis, and treatment options for this rare complication of DISH.

## Introduction

Osteophytes are bony projections resulting from abnormal osteogenic proliferation commonly seen in the degenerative cervical spine at points experiencing chronic strain. In the cervical spine specifically, osteophyte development is commonly a response to inhomogeneity in pressure on the vertebral end plate and facet joint secondary to intervertebral disc degeneration [1]. Cervical osteophytes could be caused by diffuse idiopathic skeletal hyperostosis (DISH), ankylosing spondylitis, and osteoarthritis [1]. Several terminologies have been used to denote cervical osteophytes, such as cervical exostosis, cervical arthritis, cervical arthrosis, and cervical spondylosis [2].

Cervical osteophytes are more common in middle- and older-aged groups of people; they are found approximately in 20%-30% of the elderly. Although most cases are asymptomatic, large cervical osteophytes could be associated with symptoms such as dysphagia and neck pain [3]. Cervical osteophytes compress the posterior pharyngeal wall and esophagus, causing dysphagia [4].

While the etiology is still not fully understood, various risk factors have strong correlations with patients presenting with diffuse idiopathic skeletal hyperostosis (DISH), including gout, hyperlipidemia, diabetes, and hyperuricemia [5, 6].

This article was previously presented as a conference abstract at the first Conference of Neuropedia for Students (CNS-I) on August 27, 2022.

## Case presentation

A 50-year-old male was referred to the outpatient neurosurgery clinic complaining of chronic dysphasia and neck pain since 2017. The patient felt that food or liquid got stuck in the chest or throat after swallowing. However, dysphagia was worse with solids. The pain initially started as mild discomfort but gradually increased in intensity, preventing the patient from sleeping. He also had difficulty swallowing.

The patient's medical history revealed that the patient suffered from multiple diseases, including benign prostate hyperplasia (BPH), lower back pain, gout, diabetes mellitus (DM), hypertension (HTN), cerebrovascular accident (CVA), and dyslipidemia. His surgical history showed a single surgery for an inguinal hernia. The patient was an active smoker. The patient was on antidiabetic, antihypertensive, and hyperlipidemia drugs.

Upon physical examination, the patient showed a slightly forward head position with limited neck motions in all directions. The palpation of the cervical spine indicated pain in the posterior part of the neck. The range of motion was restricted, notably in extension and lateral rotation. The eleventh and twelfth cranial nerves were normal; muscle power was 5/5; sensation was intact. The Hoffman test was negative; gait and deep tendon reflexes were normal. The patient was conscious, alert, and oriented, with a Glasgow Coma Scale score of 15/15.

**Figure 1:**
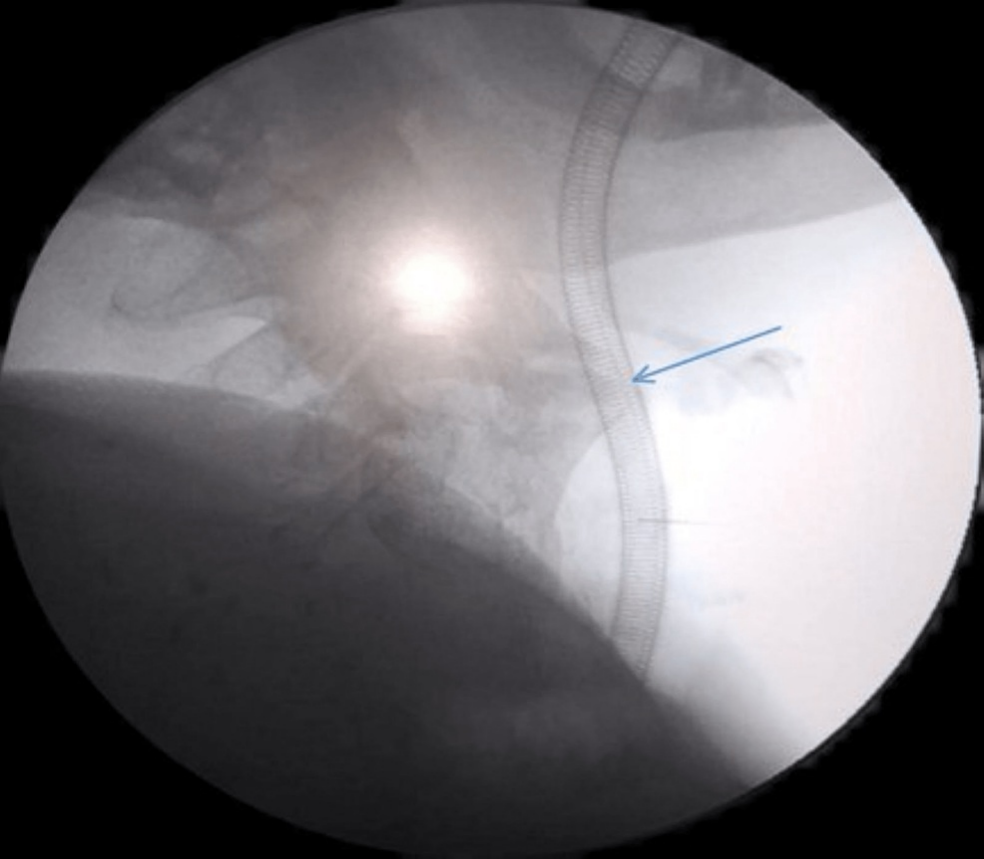
Intraoperative C-arm fluoroscopy showed anterior cervical bony calcifications at the C3-C4 level (blue arrow), which were the cause of difficulty in swallowing and discomfort.

**Figure 2:**
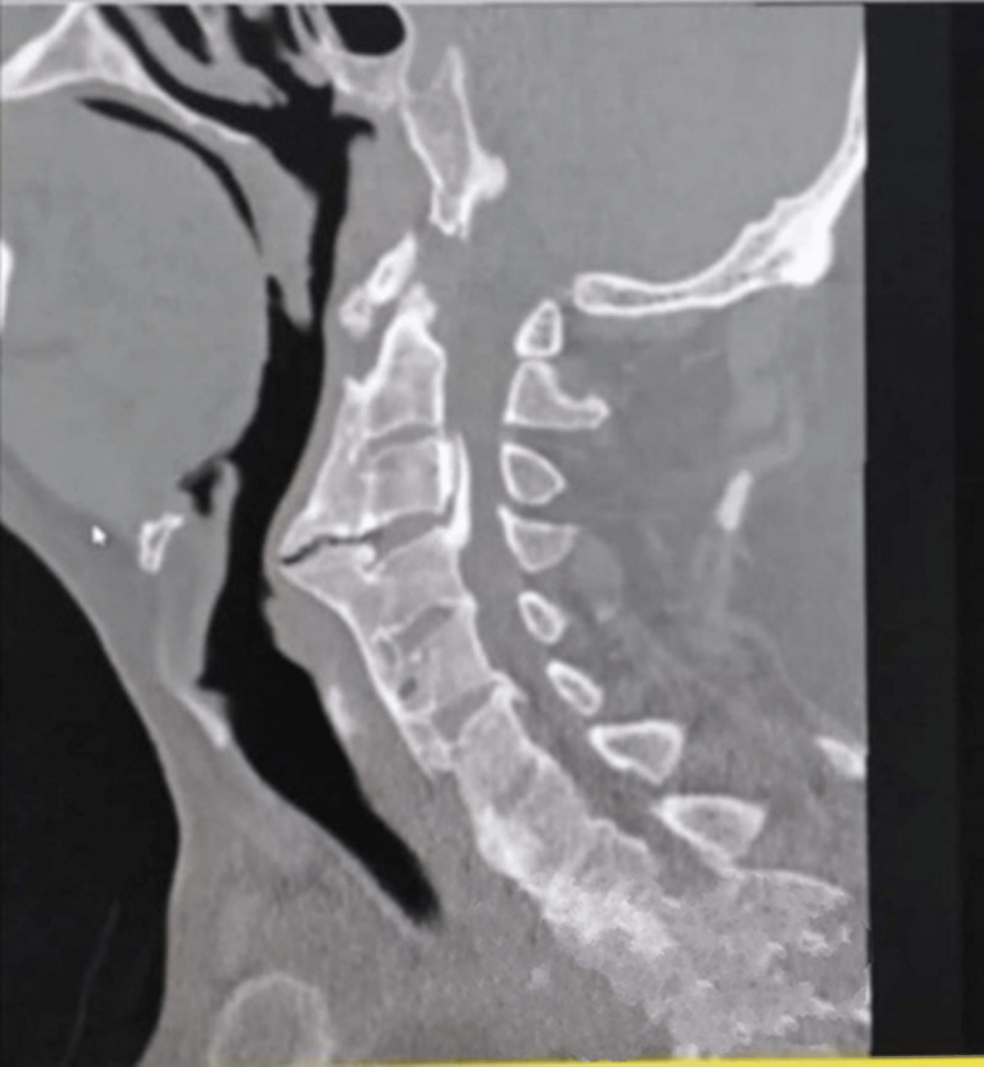
Cervical spine CT without contrast showed that the osteophyte on the anterior surface of the spine protruded and constricted the esophagus.

At that point, imaging, including C-arm fluoroscopy and cervical spine CT, revealed a huge anterior cervical calcification at the level of the C3-C4 vertebrae (Figures 1-2).

**Figure 3:**
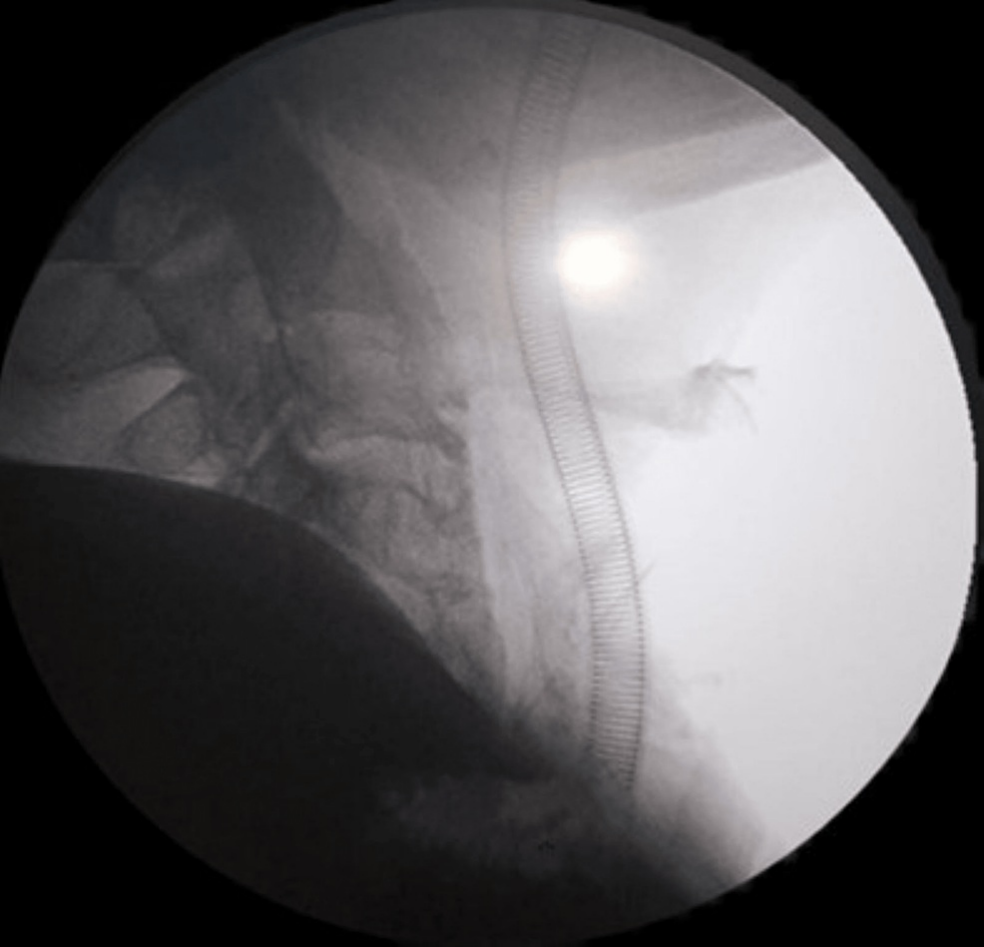
Postoperative C-arm fluoroscopy shows the resection of the osteophyte.

Operative treatment included a C3-C4 osteophytectomy. During the operation, a huge anterior C3-C4 bone osteophyte was noticed. The operation was done by skin incision at the level of C3-C4, then the direction of muscle by layers, and drilling of the osteophyte using a high-speed drill, and the incision was closed without intraoperative adverse effects. Later on, postoperative imaging revealed a complete resolution of the osteophyte (Figure 3).

The postoperative follow-up went smoothly on the ward, and the patient was discharged five days after the operation. The patient had four follow-up visits after discharge, with three-month intervals between each visit. In each visit, we observed the patient to see if there was any reemerging growth or signs of wound infection and assessed his neurological function and range of motion. Fortunately, the follow-up period was smooth.

## Discussion

Diffuse idiopathic skeletal hyperostosis (DISH) is a systemic condition characterized by ossification of the ligaments around the spinal column due to an unknown etiology. This is often asymptomatic but can cause dysphagia in rare cases [7]. In a series of 100 patients with esophageal diseases causing dysphagia analyzed by Cummings in 1946, one case was documented due to osteophytes [8]. Later in 1962, le Roux studied 1200 patients with dysphagia, in which none of the symptoms were caused by cervical osteophytes [9]. In 1960, Hilding and Tachjian reported 36 recorded cases of dysphagia due to osteophytes and presented three more of their own [10]. In 1970, Saunders reviewed 20 cases and presented two cases [11]. There has been a steady increase in reported cases of dysphagia due to osteophytes since 1980.

According to Verlaan et al., who analyzed 204 cases of dysphagia resulting from DISH, three significant patterns were noticed: (1) the major affected vertebras were C3, C4, and C5; (2) the prevalence of DISH increases with age; and (3) the male-to-female ratio was 2:1 [5].

The calcification was most likely caused by dystrophic calcification as a result of cellular damage or inflammation [12]. Trauma, infection, degeneration, or metabolic problems are all potential causes of cellular damage or inflammation in this case [12, 13]. Smoking, diabetes, hypertension, and a history of cerebrovascular accidents may have contributed to the vascular and metabolic variables that lead to calcification.

The pathogenesis of DISH is currently unknown. Diffuse idiopathic skeletal hyperostosis is characterized by flowing ossifications along the anterior spine spanning ≥4 vertebral bodies [14]. The presence of DISH has been associated with older age, male sex, obesity, hypertension, atherosclerosis, and diabetes mellitus. [14] Because the new bone forms mainly at entheseal sites, local fibroblasts, chondrocytes, collagen fibers, and calcified matrix are probably influenced by genetic, vascular, metabolic, and mechanical factors [15].

Our case is unique because osteophytes caused by DISH are most commonly asymptomatic and most commonly occur in the thoracic vertebrae [1], while our case report discusses a case in which the osteophyte occurred in the cervical vertebra (C3/C4) and the huge esophagus-type osteophyte caused chronic dysphagia and neck pain.

Esophagus-type cervical spondylosis is easily misdiagnosed or missed because of its lower incidence [16]. Although cervical spondylosis is a common disorder, dysphagia induced by osteophyte formation is uncommon. Fewer than 100 cases of cervical osteophyte-induced dysphagia have been reported [17]. This was seen in our case, in which the patient suffered from chronic dysphagia and neck pain for almost five years, and due to the low incidence, it was missed to be diagnosed.

Cervical CT revealed a massive bone osteophyte at the C3-C4 level, confirming the diagnosis of anterior cervical calcification. This imaging technique is widely regarded as the gold standard for identifying and characterizing calcifications in the spine and other areas [12]. Plain radiography and MRI, for example, may have inadequate sensitivity and specificity for this condition [12].

The management of anterior cervical calcification is determined by the severity of the symptoms and the degree of compression on the neighboring tissues. Conservative treatment for cervical osteophytes is an option, but it does not cure the underlying cause and cannot be used in cases where the osteophyte has caused unbearable symptoms. Surgical resection of cervical osteophytes is a sufficient method for treating spondylogenic dysphagia where conservative management fails to control symptoms or weight loss is observed [18]. High patient satisfaction and improvement of the quality of life are achieved with a low complication rate [19]. In this case, the osteophyte was surgically removed (C3-4 osteophytectomy) by an anterior route.

In addition to the clinical characteristics of this illness, this case emphasizes some of the possible complications connected with anterior cervical spine surgery. Although this method offers some advantages over posterior procedures, including direct access to anterior disease, less muscle dissection, and lower rates of pseudarthrosis, it also involves a risk of harm to critical tissues in the anterior neck area. These include vascular structures (e.g., carotid artery, vertebral artery), aerodigestive structures (esophagus, trachea), neurological structures (e.g., recurrent laryngeal nerve, phrenic nerve), endocrine structures (e.g., thyroid gland), and osseous structures (e.g., vertebral bodies) [20].

Some of these complications can be fatal or result in lifelong impairment if not identified and treated immediately. For example, esophageal perforation might result in mediastinitis, sepsis, or the development of a fistula. [20] Hemorrhage, stroke, or pseudoaneurysm can result from vertebral artery damage. Recurrent laryngeal nerve palsy can result in hoarseness, aspiration pneumonia, and vocal cord paralysis. [20] As a result, surgeons doing anterior cervical spine surgery must be aware of the anatomy and possible problems of this approach and utilize rigorous surgical techniques and proper intraoperative monitoring to avoid or minimize these difficulties.

## Conclusions

Cervical osteophytes that cause dysphagia are rare and difficult to identify, needing a comprehensive assessment with imaging modalities such as cervical CT. Treatment options include conservative treatments as well as invasive operations, depending on the severity of the symptoms and the compression on nearby tissues. This example demonstrates the importance of a multidisciplinary approach involving neurosurgeons, radiologists, and other healthcare professionals to provide the best cervical osteophyte inspection, diagnosis, and treatment. More research is needed to completely understand the underlying etiology, risk factors, and treatment options for cervical osteophytes. Increased healthcare practitioner knowledge will allow for earlier identification, faster action, and better patient outcomes.

## References

[1] Luo TD, Varacallo M (2023). Diffuse Idiopathic Skeletal Hyperostosis. https://www.ncbi.nlm.nih.gov/books/NBK538204/.

[2] (2023). Cervical spondylosis. https://my.clevelandclinic.org/health/diseases/17685-cervical-spondylosis.

[3] Bone RC, Nahum AM, Harris AS (1974). Evaluation and correction of dysphagia-producing cervical osteophytosis. Laryngoscope.

[4] Mashhadinezhad H, Bagheri R, Rad MF, Mashhadinezhad A (2010). A dysphagia due to anterior cervical spine osteophyte: a case report. Iran J Otorhinolaryngol.

[5] Verlaan JJ, Boswijk PF, de Ru JA, Dhert WJ, Oner FC (2011). Diffuse idiopathic skeletal hyperostosis of the cervical spine: an underestimated cause of dysphagia and airway obstruction. Spine J.

[6] Belanger TA, Rowe DE (2001). Diffuse idiopathic skeletal hyperostosis: musculoskeletal manifestations. J Am Acad Orthop Surg.

[7] Te Hennepe N, Hosman AJF, Pouw MH (2020). Dysphagia due to osteophytes of the cervical spine (Article in Dutch). Ned Tijdschr Geneeskd.

[8] Cummings GO (1946). One hundred cases of esophageal diseases‏. J Maine M A.

[9] Le Roux BT (1962). Dysphagia and its causes. Geriatrics.

[10] Hilding DA, Tachdjian MO (1960). Dysphagia and hypertrophic spurring of the cervical spine. N Engl J Med.

[11] Saunders WH (1970). Cervical osteophytes and dysphagia. Ann Otol Rhinol Laryngol.

[12] Buttermann GR (2018). Anterior cervical discectomy and fusion outcomes over 10 years: a prospective study. Spine (Phila Pa 1976).

[13] Bhatia NN (2009). Long-term outcomes and complications following anterior and posterior cervical spine surgery. Semin Spine Surg.

[14] Le HV, Wick JB, Van BW, Klineberg EO (2021). Diffuse idiopathic skeletal hyperostosis of the spine: pathophysiology, diagnosis, and management. J Am Acad Orthop Surg.

[15] Kuperus JS, Mohamed Hoesein FA, de Jong PA, Verlaan JJ (2020). Diffuse idiopathic skeletal hyperostosis: etiology and clinical relevance. Best Pract Res Clin Rheumatol.

[16] Tan HL, Luo C, Zhang R (2017). Diagnosis and treatment of esophagustype cervical spondylosis (Article in Chinese). Zhongguo Gu Shang.

[17] Davies RP, Sage MR, Brophy BP (1989). Cervical osteophyte induced dysphagia. Australas Radiol.

[18] Horkoff M, Maloon S (2014). Dysphagia secondary to esophageal compression by cervical osteophytes: a case report. BC Med J.

[19] Ruetten S, Baraliakos X, Godolias G, Komp M (2019). Surgical treatment of anterior cervical osteophytes causing dysphagia. J Orthop Surg (Hong Kong).

[20] Charalampidis A, Hejrati N, Ramakonar H, Kalsi PS, Massicotte EM, Fehlings MG (2022). Clinical outcomes and revision rates following four-level anterior cervical discectomy and fusion. Sci Rep.

